# Associations of sleep and emotion regulation processes in childhood and adolescence - a systematic review, report of methodological challenges and future directions

**DOI:** 10.5935/1984-0063.20220082

**Published:** 2022

**Authors:** Friederike Lollies, Marisa Schnatschmidt, Isabell Bihlmeier, Jon Genuneit, Tina In-Albnon, Martin Holtmann, Tanja Legenbauer, Angelika Anita Schlarb

**Affiliations:** 1 Bielefeld University, Faculty for Psychology and Sports - Bielefeld - North Rhine Westphalia - Germany; 2 University of Tuebingen, Faculty of Science, Clinical Psychology - Tuebingen - Baden -Wuerttemberg - Germany; 3 Leipzig University, Pediatric Epidemiology, Department of Pediatrics, Medical Faculty - Leipzig - Saxony - Germany; 4 University of Koblenz-Landau, Clinical Child and Adolescent Psychology and Psychotherapy - Koblenz-Landau - Rhineland Palatinate - Germany; 5 Ruhr University Bochum, LWL - University Hospital Hamm for Child and Adolescent Psychiatry - Hamm - North Rhine Westphalia - Germany

**Keywords:** Sleep, Sleep Deprivation, Emotions, Child, Emotional Regulation

## Abstract

Sleep and emotions are closely associated; however, the methodological challenges in the examination of sleep and the processes of emotion regulation in children and adolescents have not been investigated so far. Additionally, there is the demand to identify the levels of emotion regulating processes in which problematic or restricted sleep causes effect. Experimental sleep deprivation as well as prevalent sleep problems have been found to have negative influence on mental health and regulating functions. This review focuses first on the methodological protocols of the included studies. Subsequently, the results are summarized in the context of a multilevel model of emotion regulation. Thereafter, suggestions for future directions are given. Sleep problems and sleep deprivation are associated with a decrease of functional emotion regulating behavior and impaired emotion generation, and prolonged sleep enhances better mood and affect states, positive emotion expression, and faster sensory processing in response to emotional stimuli. This literature review highlights the limitations in current research, focusing on types of measurements, task characteristics, and data analysis. At the conclusion, suggestions are given for the future research direction in the field of sleep and emotion regulation in children and adolescents.

## INTRODUCTION

### Problematic sleep and the risk for mental health

The relevance of sleep to emotional and affect regulating processes has been well established^1,2^. This becomes even more important, considering that about 15%-30% of children and adolescents experience difficulties with sleep, characterized by symptoms of insomnia such as sleep onset delay or prolonged nocturnal awakenings, and poor sleep quality^3,4^. This high prevalence is concerning, as the health risk associated with problematic sleep includes the development of affective and emotional problems^1,5,6^, and shortcomings in multiple domains of emotion regulation functioning^7,8^. According to cross-sectional data, inadequate sleep patterns seem to be associated with symptoms of childhood anxiety and impulsivity^9^, higher levels of hyperactivity and more conduct and peer problems^10^. It was also postulated that persistent sleep problems in childhood predicted adulthood anxiety disorders and affective dysregulation as long-term effects^2,11^. Furthermore, studies following an experimental design have brought evidence for a direct interconnectedness of sleep and emotion regulation processes; when sleep of healthy people has been manipulated with nights of sleep deprivation, evidence was found of more negative affect states^12,13^, less positive mood, with more feelings of tension and anxiety^14^, more symptoms of depression^15^, and less happiness^16^.

Whereas the enlightening of the associations between sleep and emotion regulation in children and adolescents is necessary to develop a better understanding about their impact on developmental processes, investigation of these associations is still challenging because emotion regulation is a broadly used construct, whose operationalization as well as outcomes are poorly defined^17^.

### Emotion regulation - a multifaceted framework from developmental perspective

The process model of emotion regulation by Gross (1998)^18^ seems to be one of the most cited available theoretical frameworks to understand emotion regulation^19^. Whereas this well-established conceptualization of emotion regulation primarily focuses on intrinsic emotional response modifying processes to accomplish the individual goals, developmental aspects of emotion and affect regulation, as children’s social interaction with others are left out of consideration^20^. Especially developmental research favored a multilevel definition of emotion regulation, including different dimensions of regulation processes^21^, considering the intrapersonal, as well as social aspects of emotion regulation^22^. Within this conceptualization emotion regulation is considered as an adaptive system including physiological, attentional, emotional, behavioral, cognitive, and interpersonal levels^22,23^. However, the question of “what is regulated” per level remains open^24^. A multifaceted systemic scheme organizes the umbrella term of emotion regulation into a structured framework with an encompassing range of concrete objectives related to different levels of emotion regulation^24^ (see Table 1).

**Table 1:** Summary of levels of emotion regulation (Calkins and Fox, 2002)^22^ and related process objectives (Thompson, 1994)^24^.

Emotion regulation level	Emotion regulation processes/objectives
Physiological level	• Regulation of reactions of the nervous system • Regulation of arousal through, e.g. response inhibition • Interpretation of biological cues related to emotional arousal
Attentional level	• Shifting/redirecting attention • Behavioral distraction • Speed of processing
Emotional level	• Evaluation of positive or negative affect • Regulation of upcoming tension
Behavioral level	• Controlling the intensity of emotional reactions with consideration of environmental demands • Estimation and implementation of appropriate behavioral reaction • Fight or flight decision
Cognitive level	• Cognitive coping • Construal’s of emotionally arousing events • Reattribution of emotional content • Defense mechanisms
Interpersonal level	• Interpersonal coping • Estimation of emotional requirements of familiar settings • Selecting settings with which being emotional comfortable

Within neurophysiological processes, the activity of the nervous system to manage emotional arousal is central. Regions of the temporal cortex, particularly the amygdala is a key component of the cortical emotional processing^25^ known to show promptly responses of the nervous systems to a manifold of arousing stimuli^26,27^. The competence of e.g. inhibitory control over emotional arousal or executive cognitive functioning, growths proportional to the progress of cortical development^28^.

Governance of attentional processes is one of the first attempt of emotion regulation expected to appear during infancy and continues to be used in late adulthood^29,30^. Already young children are able to escape from emotionally arousing events through shifting their attention towards stimuli voluntarily to reduce their emotional reactivity^30^. Those regulation strategies of attention management become more complex with age and involve the internal redirection of attention, as thinking positive during distressing experience^31^ or behaviorally distraction^32^. Measurement of these attentional processes in response to emotional stimuli is somewhat difficult due to its internal character, but reaction times, as well as measuring accuracy to e.g. spatial cues with emotion eliciting content represent an opportunity to objectivate attentional processing^33^.

Whereas regulation of attention towards emotion eliciting events at young age can be governing extrinsic, e.g., through parental assistance, the intrinsic component of emotion regulation is represented by constructs of emotionally arousing events and emerge with age^24^. Such cognitive self-defense mechanisms include rationalization as well as reappraisal, which involves deliberately changing the way individual think about the meaning of an emotionally arousing stimulus or situation^32,34^. Therefore, these mechanisms are expected to result in modified personal causal attributions of affect and emotion arousing events^24^.

Next, the encoding of biological emotional cues is also an attempt to regulate emotional arousal. Biological indices for an advanced affective state are increased heart, and breathing rate or perspiration^35,36^. As an increased heart rate is the physiological response towards an external stimulus^37^ the emotional response of fear is the result of the perception process and an individual is willing to assume that fleeing will be the appropriate behavioral response^38^. Consequently, the interpretation of biological cues regulates the behavioral response.

Additionally, the access to coping strategies is an important facet of emotion regulation. When people believe they possess sufficient resources to cope with stressors, they experience a challenge response associated with positive outcomes such as mastering a challenging situation or feeling resilient^39^. In contrast, when situational demands are perceived as exceeding resources, individuals experience threat resulting in e.g. impairment of executive functioning and decision-making^40^. In early childhood material coping, such as playing with a favorite toy or listen to a radio play, as well as interpersonal coping mechanisms such as seeking (physical) proximity to caregivers under stressful situations, is common^41^. With increasing age, interpersonal coping becomes superior, e.g. peers are sought out for their expected emotional support^42^. Subsequently, individuals create their everyday life interactions, as well as their environmental life-style setting, as social relationships, work-place, family, memberships, etc., in accordance to their self-perceived needs, including emotional demands which is valued as comfortable and manageable^43^.

Finally, the process of choosing a functional expression of emotion means generating an appropriate behavioral reaction representing the individually perceived emotional arousal. For example, a careful analysis of the emotions of all parties involved in a peer conflict, combined with an insight into the negative interpersonal consequences of anger and aggression, may help a person to find a satisfactory way out of this challenging situation by trying to get others to support him or her and protest together against an unjust state of affairs instead of blindly lashing out in anger^24^.

### The gap in methodological discussion

Recently, a variety of subjective and objective measurements was used in studies to assess the association of sleep and emotion regulation. In addition, different paradigms with stimuli that address affect and emotion, such as separation scenarios or puzzle tasks, emotional images or faces, have been implemented to assess how sleep influences children’s and adolescents’ responses to those stimuli in an objective manner. These reviews addressing sleep and emotion regulation processes in children have focused on sleep and psychopathological symptoms^6^, or on general consequences of sleep loss^44^. Of course, there are reviews investigating the association of sleep and emotion in adults^1,2,45^. These have concentrated on sleep effects on individual constructs of emotion, e.g. social emotion^2^. One integrative review concerned sleep effects on levels of emotion regulation in adults^1^, but a methodological discussion component was lacking. Especially this discussion is important, because differences in methods can cause different results.

In sum, the question remains open^2^ about the influences of sleep manipulation or sleep problems on different constructs of a multifaceted concept that represents emotion regulation processes for a young population, as well as the questions of methodological challenges to evaluate the association of sleep and emotion regulation. The present review has been initiated to fill this gap and, it is intended to be a practical, methodological assistance to researchers when planning and analyzing future research on the association of particular levels of emotion regulation processes and sleep in children and adolescents.

## OBJECTIVES AND METHODS

The primary goal of this review was to initiate a systematic review focusing on the methodological protocols, thus involving different paradigms and designs of the current literature investigating the effect of sleep on levels of emotion regulation processes in children and adolescents. In detail, we began by systematically reviewing the sleep and emotion literature, including their subjective and objective outcome measures, as well as experimental tasks and their longitudinal and cross-sectional designs. Secondly, the results of the investigation are summarized and discussed in the context of the introduced processes of emotion regulation^24^. Additionally, key methodological limitations are discussed. This review concludes by suggesting some future directions for further research.

### Criteria of study selection

Inclusion and exclusion criteria are based on the recommendation of the preferred reporting items for systematic reviews and meta-analysis (PRISMA) statement^46^ (see Table 2). It should be noted, that this systematic search was not restricted to a particular study design, due to our aim to figure out the complexity as well as the heterogeneity while investigating the association of sleep and emotion regulation.

**Table 2:** Inclusion and exclusion criteria according to the PRISMA recommendation.

Inclusion criteria	
Study characteristics	- Articles were available via the chosen databases - Unpublished studies were included if they are available - Written in English - The title, abstract or keywords contained the listed search terms
Types of studies	- Experimental studies - Correlation studies - Data gathering based on parental, teacher or self-report - Objective and subjective measurement of sleep
Participants	- Families with healthy and normally developed children - Adolescent persons - 0-18 years of age - Participants are in normal physical and psychological health and without medication - Participants suffer from problematic sleep
Types of intervention	- Trials investigating the effect of experimentally induced sleep restriction or scheduled sleep manipulation - Cross-sectional and longitudinal
Types of outcome measures	- Sleep duration, quality, efficiency - Temperament, mood, emotional-responses, knowledge, problems, functioning, regulation
**Exclusion criteria**	
Types of studies	- Studies of sleep restriction omitting the investigation of emotional components - Studies, that are no longer publicly available and there was no response from the corresponding authors
Types of publication	- Book chapters, commentaries and reviews
Participants	- Studies with samples of participants older than 18 years of age
	- Samples with diagnosed psychological disorders, chronically diagnosed illness, or special medical circumstances

Regarding the chosen outcome measure of emotion regulation processes, we attempt to outline differences in assessment and study results through the span from early childhood to adolescence. Because data assessment of infants or toddler’s emotion regulation includes usually subjective, parental report, objective measures are more common in older children and adolescents^47^. Therefore, we did not restrain our search to studies including either subjective or objective methods. Furthermore, different regulation processes are assessed differently, a constraint to certain methods, such as emotional tasks making use of emotional stimuli or stress inducing paradigms, would lead to fail the aim of the present review.

Additionally, to enlighten the influence of distinct assessment and parameters of sleep on the outcome measure of emotion regulation, the authors decided to include papers with subjective and objective assessment of sleep parameters as sleep quality, efficiency and duration, as well as experimental sleep manipulation as nap deprivation in toddlers and night sleep deprivation in older children and adolescents.

### Strategy of search and sources of literature

A systematic literature search according to the recommendations of Moher et al. (2009)^46^ was conducted using the electronic databases considered appropriate for health and psychology. MEDLINE, PsychINFO, PsycARTICLES, PSYNDEX, and Google Scholar were chosen databases. Publications up to beginning of May 2021 were included. Keywords representing the part of sleep were “sleep”, “sleep deprivation”, “shortened sleep”, “sleep duration”, “sleep disturbances”, “sleep problems”, “sleep disorder”, and “insomnia”. Search terms for emotion regulation were “emotion”, “regulation”, and “affect”. To complete the search formula “toddler”, “children” and “adolescent” were added to represent the relevant age groups. Search terms were combined by the Boolean operators OR and AND. Titles, abstracts, and keywords were checked to ensure that only articles dealing with the specific terms were included. Additionally, we reviewed lists of suggestions from the search engines, and articles included in the references of the chosen documents were reviewed for their relevance.

### Collection process and study information management

**Figure 1:**
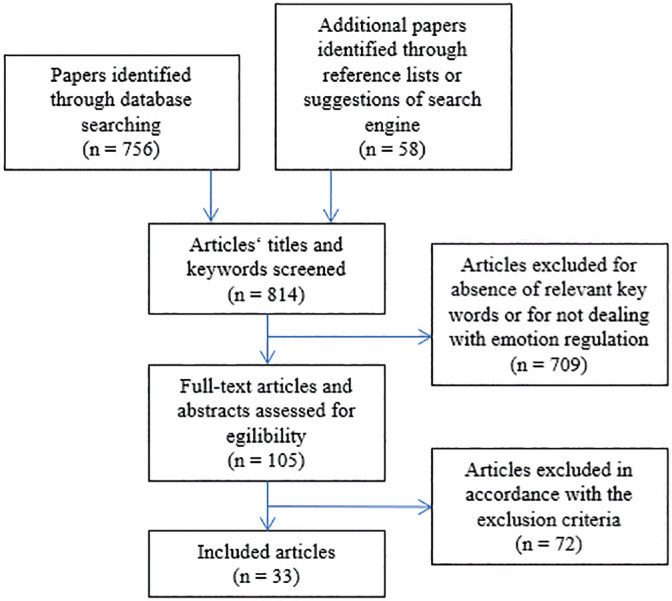
Summary of literature search and selection process.

One author (FL) screened titles and abstracts of possible research papers. As a second stage, studies were evaluated for their eligibility according to the inclusion criteria by two authors (FL, MS, process was supervised by AS). The whole paper was read and information was collected in accordance with Bonvanie et al. (2017)^48^. This structure contains: 1) aim and study designs, 2) sample characteristics, 3) details of the study, 4) outcome measures, and 5) results. Figure 1 provides the flowchart of the literature search and selection process. The resulting 32 papers were organized to their measurement to assess emotion related processes in combination with and without experimental sleep manipulation, as well as to their longitudinal character. The papers are summarized in Table 3.

**Table 3:** Summary of studies included to the review.

	Author	Subjects N (M_age in years)_	Design	Task	Stimuli	Measures	Sleep Measures	Result
1	Bastien et al. (2019)^85^	82 (2.1)	Longitudinal	-	-	Toddler behavior assessment questionnaire.	Actigraphy.	Shorter nighttime sleep duration and lower sleep efficiency at the age of 2 years predicted more anger at 3 years. Higher rates of social fear at 2 years predicted shorter day- and nighttime sleep duration at 3 years.
2	Baum et al. (2014)^69^	50 (15.5)	Between-subjects. SR: 6.5hrs of sleep SE: 10hrs of sleep for 4 nights.	-	-	Vanderbilt assessment scale, Emotion control subscale of the behavior rating inventory of executive functioning, POMS.	Sleep diary, actigraphy.	SR predicted increased levels of tension and anxiety, oppositionality, and less emotion regulation. Mood dimensions deteriorate, except depressive mood.
3	Bayes and Bullock (2020)^79^	114 (8.4)	Cross-sectional	-	-	Conner’s behavior rating scale.	Sleep disorders inventory for students-children and adolescents.	Sleep problems seem to be moderately associated to emotional distress, aggressive behavior, and impulsivity/hyperactivity
4	Berger et al. (2012)^62^	10 (2.8)	Within-subjects. Afternoon nap deprivation.	Affective response task. Unsolvable puzzle task.	11 emotional images (5 positive, 3 neutral, 3 negative). Incorrect piece in the puzzle.	Behavioral rating.	Sleep diary, CSHQ, actigraphy.	ND predicted significant more negative/less positive affect to emotional images, and duration of emotional responses during the puzzle is affected by ND.
5	Bolinger et al. (2018)^49^	16 (9,3)	Within-subjects.	Encoding and recognition task.	444 emotional images of the IAPS.	PANAS, LPP, HRD.	Stanford sleepiness scale, PSG.	After nocturnal sleep, emotional responses that are automatic as HRD increase, and cognitive emotional responses as subjective behavioral ratings and neurological activity LPP decreased.
6	Cho et al. (2017)^60^	123 (2.0)	Longitudinal	A 5 min version of laboratory temperament assessment battery. Snack delay task.	6 laboratory episodes Puppet show Clown interaction Stranger approach Stranger working Spider Robot	Behavioral ratings, ITSEA, and ECG.	Sleep diary.	Longer sleep duration predicted fewer internalizing symptoms in children showing a higher RSA.
7	Cremone et al. (2017)^52^	43 (4.6)	Between-subjects	Dot-probe task.	32 happy/neutral and angry/neutral face pairs on a screen. Trial: fixation (500ms), stimuli presentation (1000ms), probe (1100ms).	Accuracy and reaction times.	PSG measures.	No emotional attention bias following N. ND exhibit bias to negative and positive stimuli. Greater SWA during N predicted faster responding to emotional stimuli.
	**Author**	**Subjects** **N (M_age in years)_**	**Design**	**Task**	**Stimuli**	**Measures**	**Measures-Sleep**	**Result**
8	Dagys et al. (2012)^68^	47 (13.1)	Within-subjects. SR: 2hrs of sleep SE: 8.5hrs of sleep for 2 nights.	-	-	PANAS-C, children’s morningness-eveningness preferences scale.	Duke structured interview for sleep disorder, sleep diary, actigraphy.	SE predicted more positive affect, positivity. No difference concerning negative affect between SE and SR. Evening as well as morning chronotypes displayed less positive affect after SR.
9	DeLeon and Karraker (2007)^65^	41 (0.7)	Cross-sectional	-	-	Revised infant temperament questionnaire, Infant/Toddler symptom checklist.	Infant care diary.	Rhythmic and adaptable infants took longer naps and slept more at night. Distractible children took shorter and more frequent naps.
11	Foley and Weinraub (2017)^82^	1057	Longitudinal. Assessment took place at the age of 54 months in grade 1, 3, and 5.	-	-	Generated questionnaire for feelings, risky behavior and emotional regulation. Child behavior checklist. Children’s depression inventory.	CBCL.	Early sleep problems predicted anxious-depressed symptoms in the middle childhood, a higher rate of emotional reactivity in the preadolescence. Gender differences in temporal development of sleep and emotion problems exist.
12	Gregory and O’Connor (2002)^81^	490 Assessments from 3 to 15 years of age.	Longitudinal	-	-	CBCL.	CBCL.	Early sleep problems at 4y predicted depression/anxiety, attention problems, and aggression in adolescent age No evidence of early depression/anxiety symptoms predicting later sleep problems.
13	Gruber et al. (2012)^77^	33 (8.6)	Within-subjects. SR/SE: 1hr later/earlier to bed for 5 nights.	-	-	Connors’ global index-teacher.		SE predicted significant lower emotional lability and restless-impulsivity.
14	Gruber et al. (2020)^80^	122 (8.6)	Cross-sectional	-	-	CBCL.	CSHQ, actigraphy.	Children scored above the cut-off of the CSHQ had more emotional problems. Data is in consent with the subjective sleep data.
	**Author**	**Subjects** **N (M_age in years)_**	**Design**	**Task**	**Stimuli**	**Measures**	**Measures-Sleep**	**Result**
15	Han (2014)^66^	14 (4.8)	Within-subjects. Afternoon nap deprivation.	Affective response task.	34 emotional images with appropriate auditory stimuli (8 strong negative and positive, 8 weak positive and negative). Trial: 11s, fixation (2s), cue to attention (2s), stimuli presentation (7s).	fEMG.	Sleep diary, actigraphy.	ND predicted greater emotional responses to strong negative and positive stimuli. No change in affective responses to weak stimuli. Emotional responses to emotional pictures were lower after the N.
16	Kouros and El-Sheik (2015)^72^	142 (10.7)	Cross-sectional	-	-	Daily mood report, Personality inventory for children.	Actigraphy.	Sleep latency, efficiency, mood and behavioral problems were found to be interconnected significantly.
17	Lo et al. (2016)^55^	56 (16.6)	Between-subjects. SR: 5hrs of sleep SE: 9hrs of sleep for 7 nights.	-	-	PANAS.	Karolinska sleepiness scale, Pittsburgh sleep quality index, actigraphy, PSG.	SR predicted a decrease of positive affect with a lowest point at the last day of sleep restriction. No significant change of negative mood through sleep restriction.
18	McMakin et al. (2016)^67^	48 (13.3) 16 (14.5)	Within subjects. SR to 4hrs of sleep for 2 nights SR to 6hrs of sleep on 1 night, and 2hrs of sleep on the second night. SE: 10hrs of nocturnal sleep.	Peer conflict task. Auditory valence identification task. Affective response task.	Individual real-life disagreements. 42 emotion eliciting sound clips (14 positives, negative, neutral). Trial: 15s, orientation (1s), stimuli presentation (6s), rating interval (8s).	Behavioral rating, accuracy, reaction times, pupillography and by subjective self-report.	PSG	SR predicted more self-reported and objective measured negative affect. SR predicted less positive affect in study 1, not in study 2. Negative affective behavior was significant higher after sleep restriction.
19	Miller et al. (2015)^61^	12 (2.8)	Within-subjects. Afternoon nap deprivation.	Unsolvable puzzle.	Incorrect piece in the puzzle.	Rating observation.	Sleep diary, CSHQ, actigraphy.	ND predicted less skepticism, and negative self-appraisal. ND predicted more physical self-soothing, perseveration, and tenancy.
20	Raynolds (2017)^73^	20 (15.7)	Within-subjects. SE: 1hr earlier to bed for 5 nights.	Online social interaction task. Paced auditory serial addition task.	Getting to know an unknown person. Fast calculating.	Daily mood questionnaire. The self-assessment Manikin. Computer based linguistic inquiry and word count, facial expressions valence.	Sleep diary, actigraphy.	SE predicted more negative facial expression and higher levels of facial expression variability. No change in emotional language, subjective report of emotion regulation, persistence or task performance.
	**Author**	**Subjects** **N (M_age in years)_**	**Design**	**Task**	**Stimuli**	**Measures**	**Measures-Sleep**	**Result**
21	Reddy et al. (2017)^57^	42 (14.8)	Between-subjects. SR: 2hrs later to bed. SE: 9.5hrs in bed.	Emotion reactivity and regulation task.	40 emotional images (8 positive and neutral, 24 negative). Trial: 18s, 10s stimulus presentation, 8s rating interval.	PANAS, State-trait anxiety inventory for children. Emotional reactivity and ER was assessed by subjective valence, intensity/arousal, and reappraisal ratings.	Epworth’s sleepiness scale, BEARS sleep screen, sleep diary, actigraphy.	SR predicts subjective decrease of positive affect and increase of state and trait anxiety. No change in emotional reactivity and regulation.
22	Ross and Karraker (1999)^63^	40 (1.3)	Between subjects. 20 subjects were assessed before The other 20 subjects were assessed after their regular nap.	Rieser-Danner’s plexiglas barrier task. Parts of the Laboratory temperament assessment battery. Ainsworth’s strange situation procedure.	5 Stressing episodes, Toys in jar, Remote-controlled toy approach, Maternal separation, Attractive toy, Mother busy.	Behavioral rating. Infant behavior questionnaire.	-	Fatigue sensitizes infants to certain stressors instead of simply increasing irritability and interferes with infants’ coping responses. Exhausted children exhibited a higher degree of fatigue frustration.
23	Rubens et al. (2017)^78^	285 Assessments from 3^rd^ to 5^th^ grade	Longitudinal	-	-	Children’s emotion management scales, Pediatric anxiety scale of the patient-reported outcomes measurement Information system, Short mood and feelings questionnaire, Affective reactivity index, Self-report scale for deviant behavior, Self-reported reactive/proactive social behavior.	Sleep quality was assessed by subjective 4-item child self-report scale.	Better sleep quality predicted lower self-reported emotional and behavioral problems. Regarding gender effects girls scored higher on the anxiety scale and lower on irritability, delinquency engagement and reactive aggression.
24	Saenz et al. (2015)^84^	47 (1.6)	Longitudinal	-	-	BITSEA.	Sleep diary, actigraphy.	In girls, shorter sleep duration at the age of 3 months predicted significant more externalizing problems at the age of 18 months.
25	Schumacher et al. (2017)^53^	19 (3.8)	Between-subjects. SR: 3hrs later to bed for 1 night.	A go/no-go task. Unsolvable puzzle.	No-go trial (pig). Incorrect piece in the puzzle.	Accuracy, rating observers.	Sleep diary, actigraphy.	No significant effects of sleep restriction on response inhibition or self-regulation. Interaction effect of response inhibition and sleep condition on adaptive self-regulation and maladaptive self-regulation.
	**Author**	**Subjects** **N (M_age in years)_**	**Design**	**Task**	**Stimuli**	**Measures**	**Measures-Sleep**	**Result**
26	Settineri et al. (2010)^71^	529 (17.1)	Cross-sectional	-	-	Mood was assessed by subjective measurement with an 8-item scale.	TST, napping and sleepiness was assessed by subjective measurement with a 4-item scale.	Well-being at awakening had a negative correlation with sadness, apathy, anhedonia, and pessimistic thoughts. Well-being at awakening was positively correlated with TST, negatively with afternoon naps and daytime drowsiness.
27	Short and Louca (2015)^70^	12 (16.2)	within-subjects. SR: 36hrs of wakefulness	-	-	POMS - short form.	Sleep diary, Karolinska sleepiness scale, actigraphy, PSG.	Dimensions of mood significantly deteriorate during a night of sleep restriction. Increased anxiety in females but not in male participants after sleep restriction. Only girls reported an increase of depressive mood in response to SR.
28	Soffer-Dudek et al. (2011)^56^	94 (10.5 at the 1^st^ assessment)	Longitudinal	Balloons task.	Faces on balloons showing different emotional expressions.	Accuracy on judgments.	Sleep diary, actigraphy.	More night awakenings predicted less task performance on the face-emotion processing task.
29	Troxel et al. (2013)^76^	776 Assessments at 1, 6, 24, and 36, and 54 months.	Longitudinal	-	Neutral parent-child interaction at home was videotaped for 15 minutes during the visit.	Negative emotionality was behavioral rated by researcher.	CBCL-parent and teacher version.	Early sleep problems and negative emotionality predicted later internalizing behavior.
30	Vaughn et al. (2015)^58^	62 (4.1)	Cross-sectional	Denham’s emotion knowledge task.	Faces showing different emotional expressions.	Emotional knowledge was rated on the documented subjects’ ratings during the task.	Sleep diary, actigraphy.	Sleep duration had positive correlations with emotional knowledge.
31	Vriend et al. (2013)^54^	32 (9.8)	Within-subjects. 1hr SE/SR for 4 nights.	Affective response task.	33 emotional images	Subjective affect rating on visual analogue scales.	Child’s pictorial sleepiness scale, CSHQ, Sleep evaluation questionnaire, Epworth sleepiness scale, actigraphy.	SR predicted less positive affective response and poorer parental reported ER. No change in negative affect responses or ER.
32	Wang et al. (2019)^83^	1625 Assessments from 5 to 17 years of age	Longitudinal	-	-	Dysregulation profile of the CBCL.	CBCL	Persistent sleep problems, measured over a span from five to 17 years found to contribute to a ten-time increased risk for developing regulatory difficulties.
33	Weissbluth (1981)^64^	60 (0.6)	Cross-sectional	-	-	Carey infant temperament questionnaire.	Sleep interview.	Significant negative correlations between TST and mood, adaptability, rhythmicity, withdrawal, and persistence. Children described as “difficult” had shorter sleep duration than “easy” children.

Abbreviations: Bedtime issues, Excessive daytime sleepiness, Night awakenings, Regularity and duration of sleep, Snoring (BEARS); (Brief) infant-toddler social emotional assessment (B)ITSEA); Child behavior checklist (CBCL); Children’s sleep habits questionnaire (CSHQ); Electrocardiography (ECG); Emotion regulation (ER); Facial electromyography (fEMG); Heart rate deceleration (HRD); International affective picture system (IAPS); Late positive potential (LPP); Napping (N); Nap deprivation (ND); Polysomnography (PSG); Positive and negative affect schedule (for children) (PANAS (-C); Profile of mood states (POMS); Respiratory sinus arrhythmia (RSA); Strengths and difficulties questionnaire (SDQ); Sleep extension (SE); Sleep restriction (SR); Slow wave activity (SWA); Total sleep time (TST).

It should be mentioned, that during the literature collection and selection process it was conspicuous, that almost 95% of the originally 814 studies were rejected because they were not eligible for the review. Reasons for that high rejection rate were, e.g., that despite defining a young population without appreciable health related limitations in the search formula, also a great number of studies with adults and participants suffering from chronical illness were found in the result pool of the literature search process.

### Missing data

If full-texts were missing, correspondence authors were contacted once via mail and asked to provide the full text, if those papers were not provided, they have been excluded from further analysis in this review (n=2).

## RESULTS

### Effect of sleep on neurophysiological processes linked to emotion regulation

In order to assess the effect of sleep on emotion related neurophysiological activity researchers utilized EEG measurement.

*Cortical activity.* Bolinger et al. (2018)^49^ were interested in recordings of late positive potentials (LPP), which are recognized to be a neurophysiological marker for emotion regulation in children and is modulated by conscious cognitive processes^50^. They assessed the processing and recognition of neutral, as well as emotional visual stimuli. Two points of assessment were planned in a sample of children; first, an encoding phase included affective ratings of pictures with emotional content, second the recognition phase took place ten hours later, in the evening following encoding for the “no sleep” condition, and on the next morning for the “sleep” condition. The accuracy of recognition increased significantly after sleep. Thus, sleep seemed to enhance stimuli processing in a way that may preserve a person’s autonomic reactivity. It is striking, that while be tested in the evening (no-sleep condition), participants achieved better recognition accuracy to pictures with negative emotional content compared to be tested after sleep.

Researcher also implemented facial emotional stimuli to investigate the effect of napping on behavioural responses to emotion eliciting stimuli in pre-school children^51^_._ Cremone et al. (2017)^52^ utilized EEG recordings during a nap to assess the slow wave activity (SWA) reflecting neocortical oscillations, which contribute to emotional processing. During measurement of attention bias to emotional stimuli, the allocation of attention toward or away from emotional stimuli was assessed. Within the task, children had to click on the right or left button of the mouse to indicate the location of the stimulus as quickly and as accurately as possible. In contrast to the findings in school-aged children, accuracy and reaction times in pre-school children for negative and positive affective stimuli did not differ between the nap conditions. However, being tested before sleep was associated to greater attention bias. There was not a significantly difference for positive or negative trials. Furthermore, results indicated a greater slow wave activity (SWA) while napping was associated with a faster response to the stimuli.

Those results support the assumption that sleep seems to enhance cortical activity, and cognitive processes in school-aged children^49^, as well as the automatic direction of attention in response to presentation of emotional stimuli in pre-school children^51^. It would be very interesting to get more detailed insight into discriminating power of the emotional category of stimuli on neurophysiological reactions towards sleep deprivation in children.

### Effect of sleep on attentional processing linked to emotion regulation

Whereas the effect of sleep on attentional regulation processes as defined by Thompson (1994)^24^ were not assessed in the included studies, measures as accuracy and speed of attentional processing are summed up.

*Response inhibition*. In order to investigate the effect of sleep on attentional processing, sleep deprived toddlers^53^, children^54^, and adolescents^55^ were investigated on their response inhibition. During a go/no-go task the subjects’ accuracy to inhibit the button response in the case of the presented no-go stimulus was assessed. No main effects of sleep deprivation on response inhibition were reported after one night going to bed three hours later than usual^53^ and after one hour of night sleep extension or restriction^54^. Contrary to the findings in children^53,54^, the reaction times and accuracy in discriminating behaviour in adolescents deteriorated significantly after night sleep deprivation and did not re-establish to the baseline performance after two nights of recovery^55^.

An additional study looked for indication of the effects of sleep quality on emotional information processing in early adolescents^56^. To meet this goal, subjects had to respond via mouse-click to gender or to particular positive and negative emotions represented by faces on balloons which arose from the bottom of a computer screen. More night awakenings and lower sleep efficiency were found to predict only lower success on the face-emotion task; accuracy in responses to gender trials seemed to be unaffected. These results demonstrated that emotion information processing change as a function of night awakenings and sleep efficiency.

### Effect of sleep on reasoning processes linked to emotion regulation

*Cognitive reappraisal and emotional reasoning.* To assess the relation between sleep restriction and reasoning processes in response to emotion eliciting stimuli belonging to the IAPS, Reddy et al. (2017)^57^ assessed adolescent’s cognitive reappraisal techniques as distancing from the reality of the picture, thinking about improvement, or creation of a positive explanation. According to their results, one night of sleep deprivation had no main-effect on adolescent’s ability generating reappraisal statements as well as on their efficacy in regulating negative emotions. Contrasting results were presented by another study investigated the association of sleep parameter and emotional reasoning^58^. During the Denham’s affect knowledge task, children had to name and reason about emotion expressions represented on images^59^. Whereas sleep latency and efficiency had no significant correlation with emotion cause score, the parameter of sleep duration had positive correlations with the ability to reason about emotions^58^.

### Effect of sleep on biological cues linked to emotion regulation

Evidence for associations between sleep and affect was also found in physiological measures.

*Heart rate.* To assess the internal biological cues of emotion regulation, Bolinger et al. (2018)^49^ recorded the participants’ heart rate deceleration (HRD) in response to emotion eliciting stimuli. In contrast to the already described LPP, the HRD response to emotional stimuli decreased in the case of wakefulness. Testing after night sleep led to an increase in the HRD response to pictures with negative emotional content, whereas the HRD seem to be unaffected in case of neutral stimuli^49^.

*Parasympathetic activity.* Cho et al. (2017)^60^ investigated the association between sleep duration and emotion regulation problems in toddlers. To assess the regulation capacity, children participated in a series of short, mildly stressing, social interaction tasks. Parents reported their children’s regulatory behavior responses and, for parasympathetic reactivity, the respiratory sinus arrhythmia reactivity (RSA) was assessed. The authors report evidence of increased RSA reactivity to any interaction episodes, in combination with longer sleep duration and less internalizing symptoms. Longer sleep duration predicted fewer internalizing symptoms in children showing a higher RSA reactivity to the fear-eliciting stimuli. Subjects with shorter sleep durations showed decreased parasympathetic response, which is associated with less capacity for regulation^60^.

### Effect of sleep on coping processes linked to emotion regulation

From research in nap deprived children, it was also reported that toddlers react differently depending upon whether they are sleepy or well-rested.

*Self-regulation.* To assess coping processes, children were confronted with an age-appropriate, but unsolvable puzzle either one hour after their habitual nap or one hour after the habitual nap would normally have occurred (nap vs. nap deprivation)^61,62^. One minute after all pieces, except the incorrect one, were successfully placed, children were encouraged to finish. After nap deprivation, perceived scepticism about the piece would match, decreased. Physical self-soothing, as repetitive bodily-directed behaviors, and focussing on the piece that would not fit, thus perseverance and tenancy to complete increased after nap deprivation. Additionally, negative self-appraisal, as discrediting the competence to solve the puzzle in the unsolvable puzzle task, and display of confusion to the challenging situation, decreased after nap-deprivation^61,62^.

Another study found sleep deprivation had no direct effects on self-regulation strategies in three years old children^53^. Though, a mediating effect of response inhibition was assumed. It was reported that children with better response inhibition before sleep restriction were more likely to use adaptive self-regulation strategies, while poor response inhibition predicted increased use of maladaptive self-regulation strategies in response to the unsolvable puzzle task after sleep restriction.

To examine the effect of fatigue on infants’ emotional coping, visiting and assessment of children took place 1 hour after the infant’s regular nap would have occurred (nap deprivation) or actually occurred (nap condition)^62^. Another study used the time point of mother’s expectation of her infant to be awake or the time when the infant’s habitual morning or afternoon nap started to assess differences between alert and fatigued children^63^. The emotional stimuli included five mild stress inducing episodes. To assess children’s frustration toleration, infants were encouraged to play with toys placed in an unopenable jar. Fatigued children looked more often to the experimenter and were less persistent in exploring the jar. While separated from their mothers’, fatigued children were more focused on proximity seeking, and fatigued children showed also more self-soothing behaviour during an episode of prohibiting to play with an attractive toy. This study supplied evidence that, in response to different stressors, fatigued infants are more emotionally reactive and less mature in their emotion regulation capacities than rested infants.

These findings are supported by cross-sectional research by Weissbluth (1981)^64^. He found significant negative correlations between total sleep duration and infants’ resilience. Furthermore, children described as “difficult” were negative in mood, less adaptable, approachable, and rhythmical and had shorter total sleep times per day, than “easy” children. “Difficult” children also had a higher level of activity and lower sensory thresholds than “easy” children. This is in line with another study^65^, with the additional finding that infants, described as rhythmic and adaptable, also took longer naps than distractible children did. Furthermore, infants described with persistent night awakenings were described as displaying more maladaptive stress-coping, as dysregulation behavior, e.g. higher levels of separation distress, than was so with continuous sleepers.

### Effect of sleep on emotion expression

According to Thompson (1994)^24^, emotion regulation encompasses generation of appropriate emotional expressions. Therefore, upcoming emotions and affects have to be processed and appropriate behavior has to be generated. Within the following paragraph the effect of sleep on the emergence of these affective responses will be summed up.

*Affective response ratings*. Focus has been on the effects of experimental sleep deprivation on affective responses to emotion eliciting stimuli. Therefore, sleep deprived and control subjects were compared on their affective arousal after confrontation with images or sounds representing pleasant, unpleasant, or neutral stimuli. To evaluate emotional responses of infants after nap restriction, emotion assessment was implemented at home^62^. To observe and quantify the children’s emotion expressions towards pictures from the International Affective Picture System (IAPS) on a screen, researchers analyzed the videotaped task sessions. Nap restriction contributed to more negative affect in response to neutral pictures and conversely positive pictures induced fewer positive reactions^62^. A comparable task was implemented in pre-school children^66^. The assessment contained strong and weak emotional visual stimuli of the IAPS, which were paired with an appropriate auditory stimulus. Affective responses were assessed by facial electromyography (fEMG). After nap-deprivation, results also demonstrated greater affective responses to strong negative and positive pictures. No sensitivity was reported of affect states to weak stimuli after nap-deprivation. Affective responses of nocturnal sleep restricted school-aged children were rated via self- and parental report after presentation of the IAPS stimuli^54^. Short sleep condition predicted less positive affective responses and more problems in emotion regulation. The study by Bolinger et al. (2018)^49^ also made use of IAPS stimuli and they assessed the subjective emotional response by asking their participants directly for their valence ratings of the stimuli after first presentation and after a recognition session following the night sleep or in the evening (sleep vs. wake condition). Whereas the valence rating leaves unchanged in the wake condition, in the sleep condition the negative valence rating of negative stimuli decreased from encoding to recognition session and neutral stimuli were rated as more negative^49^. One study protocol included the affective response task with only auditory stimuli^67^. During the task, adolescents were confronted with short sound clips. Measurement was based on subjective reports, as well as objective pupillography assessment. As reported in the visual stimuli studies, negative affect rating was significant higher in the restriction group and positive affect rating was lower after sleep restriction. Additionally, the puzzle task was implemented to evaluate the outcome measure of affective responses^62^. Nap restricted children had shorter positive emotion responses; they expressed less joy and pride after solving the puzzle. Without their afternoon nap, children also showed longer negative emotions, in particular worry and anger, when faced with an unsolvable puzzle^62^.

Another study displayed significant differences in subjective reports of positive affect between sleep extended and restricted adolescents^68^. Adolescents scored higher for positive affect when they were in the rested condition. No difference concerning negative affect was found between sleep conditions. Regarding participants’ chronotype, evening as well as morning chronotypes displayed less positive affect if they were sleep deprived. This result is congruent to the finding of a steadily decrease of positive affect during the seven-day period of sleep deprivation^55^. Data for negative affect showed no significant change due to sleep restriction. This suggests that subjective positive affect is more sensitive to sleep deprivation than subjective negative affect. Overall, studies of the effects of sleep on the affective responses to emotional stimuli reveal a rather heterogenous picture, reflecting the complexity of physiological as well as cognitive and behavioral processes of emotion regulation.

*Mood dimensions.* To assess different mood dimension, the profile of mood states with mood describing adjectives, was completed after sleep restriction in adolescents^69,70^. Whereas Baum et al. (2014)^69^ used the POMS only twice, after a baseline and after sleep restriction week, in the study by Short and Louca (2015)^70^ the (POMS)-short form was completed every two hours during the night of strict sleep deprivation. Participants of Baum et al. (2014)^69^ reported increased levels of different mood dimensions, except depression. The contrary findings of Short and Louca (2015)^70^ revealed that all dimensions of mood, inclusive depressive mood significantly deteriorate after sleep deprivation, whereas anxiety was found only in females, and depressive mood only marginally in male participants.

A short telephone inquiry was given to adolescents, to investigate the relationship between their mood states in the morning and their night sleep^71^. A good sleep quality was positively correlated to a better mood in the morning and negatively correlated to daytime drowsiness. Results were comparable to those in young children^72^. Children with longer sleep latency and low sleep efficiency had a decline in positive mood for the next day, which predicted higher sleep activity during the following night and longer sleep latencies again. Subjects showing longer sleep latencies also had an averaged negative mood stretching across the seven days of assessment, which was associated with higher levels of internalizing and externalizing symptoms in general.

While previous studies have investigated the emergence and association of positive or negative emotional states and sleep manipulation or sleep parameter, other authors have been focused on how emotions are expressed during social interaction.

*Social interaction.* Responses to tasks of social interaction seemed also to be sensitive to sleep deprivation^67^. By that method, individual recent disagreements with friends were sorted by their relevance. During the visit at the experimental laboratory, participants’ real-life friends were invited and asked to discuss one of the two most highly rated conflicts with the participants. Behavior during the task was rated by a researcher on facial expressions and on verbal content, and these summary scores of negative and positive affect behaviors were included in the analysis^67^. Observed negative affective behavior, such as conflict withdrawal and dominance during the peer conflict task, was significantly higher after sleep restriction.

The implemented task adopted in the study of Raynolds (2017)^73^ distinctly differed to those already described^67^. Within this study, the association of typical or extended sleep and emotion regulation was investigated. From a non-manipulated session, the task protocol consisted of adolescents getting to know an unfamiliar person for five minutes via an iPad. Thereafter adolescents were briefed that the next unfamiliar person had lost the phone, thus the subjects’ waiting time could be used to complete the Paced auditory serial addition task (PASAT)^74^. This task required participants to sum numbers sequentially as they appeared on the computer screen, and was designed to increase frustration and negative mood. After the task, participants did not have time to recover from the frustrating task before beginning the manipulated social interaction task, with the instruction to make the other person, who had lost the phone, feel better. Sleep extended adolescents had more negative facial expression and higher levels of facial expression variability than the typical sleep group throughout the manipulated task. Emotional language regulation, persistence, and the PASAT score did not achieve statistically significant effects.

In order to evaluate children’s negative emotionality, representing a predictor for being less confident and more vulnerable while faced with either positive or negative circumstances^75^, the mother-infant dyad was observed at a home visit during normal interaction^76^. Children with high scores for negative emotionality at the age of 6 months had more internalizing problems at the age of 54 months, when having had more sleep problems at the age of 36 months. Additionally, teachers were asked to value emotion regulation behavior of school-aged children to assess the influence of sleep manipulation^77^. The emotional outcome scores of emotional lability and restless-impulsive behavior improved after sleep extension, whereas these measures deteriorated in children experiencing sleep restriction. The finding that sleep deprivation affects facets of emotion regulation and oppositionality in a social context is supported by parental and self-report in adolescents^69^.

Whereas parental reporting is necessary while assessing data of infants and toddlers, school-aged children can give ratings on subjective measures on their own^78^. Better sleep quality was associated with less emotional and behavior problems^79^. Regarding gender effects, results indicate that girls score higher on the anxiety scale and lower on reactive aggression. Furthermore, emotional dysregulation was rated as low when children rate their sleep quality as high and young children’s dysregulation was rated high due to continues night awakenings. Thus, dysregulation seems to be sensitive to sleep parameters in infants^64^ as well as in school-aged children^78^.

The positive correlation between good sleep and emotion regulation ratings reached significance in the correlational studies including subjective measurements of sleep and affect in children and adolescents^64,65,78-80^. Because it is often asked if results regarding emotional outcome measures differ in consequence of making use of whether subjective or objective sleep measurement, one study displayed that parental reported presence of children’s sleep disturbances is a reliable predictor of objectively assessed inappropriate sleep schedules^80^. Unfortunately, results of these studies cannot investigate the interconnectedness of temperament, emotion regulation, and sleep, because assessment of affective constructs was at a single point of measurement. To assess the diverse mutual developmental trajectories of sleep and emotion regulation longitudinal research is needed.

### Interconnectedness of sleep and development of emotion regulation competence

Within their longitudinal study, Gregory and O’Connor (2002)^81^ were interested in changes of sleep and behavioral problems over the period of childhood. Results suggest that early sleep problems predict behavioral problems, in particular emotional problems, in later lifetime. However, no evidence was found for early depression, anxiety or aggression symptoms predicting more sleep problems in mid-adolescence. Further, no differences between the sexes were detectable. Foley and Weinraub (2017)^82^ researched the topic of sleep and emotional adjustment in children, and found more early sleep problems predicted more anxious-depressed symptoms in the middle childhood in both boys and girls, and this was found to be associated with higher rates of emotional reactivity in the preadolescence. In contrast to Gregory and O’Connor (2002)^81^, gender differences were found. For boys, earlier anxious-depressed symptoms predicted more problematic sleep in the preadolescence; and more negative affective temperament in early childhood was correlated with more sleep problems and anxious-depressed symptoms at all points of measurement. For girls, more early sleep problems predicted less social competence in school and this was associated with more anxious-depressed symptoms in preadolescence. Additive, higher levels of sleep problems in the middle childhood in girls predict higher levels of emotional reactivity in preadolescence. The interconnectedness of sleep and emotional problems is also displayed by a longitudinal research based on parental-report data^83^. According to the published results, it was concluded that sleep problems and problems in emotion regulation are strongly associated in their development over time, and those participants suffering from persistent sleep problems have a 10 times increased risk to develop problems with emotion regulation^83^.

To overcome the limitation of exclusive subjective sleep measurement, a study in young children implemented actigraphy for five nights when infants were three months of age^84^. Emotional problems were assessed by subjective parental reports when infants were 20 months. Regression analysis of externalizing-, internalizing-, and dysregulation problems, sleep efficiency and sleep duration led to no detectable main effects in boys. In girls, shorter sleep duration at the age of 3 months predicted significantly more externalizing problems at the age of 18 months. Whereas the finding of existing gender differences was incongruent to another longitudinal study^81^, longitudinal research of Foley and Weinraub (2017)^82^ supported an association of insufficient sleep in infancy and later affective problems in girls, and in contrast with Saenz (2015)^84^, this was also found in boys. Differences might be explained by the source of data; parental report of sleep^81,82^ may be not associated with actigraphy assessment of sleep^84^. A further longitudinal study includes objective sleep measures in toddlers to investigate the mutual dependence of sleep and emotional outcomes^85^. They display that short sleep duration and low sleep efficiency at age two are associated to more frustration and anger one year later. There were also indices for high rates of social fear at the age of 2 being associated to shorter day- and nighttime sleep duration at measurement one year later. Nevertheless, it may be informative to include actigraphy standardly in future longitudinal research on sleep and affect and emotion.

### Manipulation of sleep

To give a short insight to the differences in schedules of sleep manipulation we added this section to our review. This could be understand as hint for upcoming research, because differences in scheduling sleep for experimental research of emotion regulation could lead to different outcomes. However, a full discussion of this relationship will go beyond the scope of the review.

In sum 14 studies implemented sleep manipulation in their studies (for a detailed overview about the different protocols of sleep manipulation, they are summed up for the age groups of infants and young children, school-aged children, and adolescents in the Appendix).

Five studies implemented sleep restriction protocols or nap restriction in young children. Except one^51^, all of them implemented a sleep stabilization period of five or seven nights before sleep manipulation. Four studies restricted the afternoon nap to assess the effect of sleep loss on emotional reactions^51,61,62,66^. One study implemented a form of night-sleep restriction in young children^53^. Regarding the points of measurement, testing took place after respectively the normal and the sleep or nap restriction condition. Whereas Cremone et al. (2017)^52^ implemented the dot probe task, three studies included the unsolvable puzzle task to assess emotional regulation in toddlers. The assessment battery of Berger et al. (2012)^62^ as well as Han (2014)^66^ included emotional stimuli of the IAPS and the IADS.

In school-aged children, two studies implemented experimental sleep restriction. They implemented a stabilization period over four or six nights^80^. Within this period, children went to bed as they normally do. After stabilization, children were randomly assigned to the sleep restricted or sleep extended condition, with bedtimes one hour later or earlier than usual. Blinded teacher rated children’s emotional lability and restless-impulsivity, and the Conner’s global index-teacher was completed on the day after sleep stabilization period (baseline) as well on the last night of sleep manipulation period. Vriend et al. (2013)^54^ mixed objective (tasks) and subjective (questionnaires) measurements to assess emotion.

With regard to adolescents, five studies applied a stabilization period, which ranged from three nights^55^ to one week^70^. Instructions in the stabilization period were individual or normal, self-selected bedtimes^68,69^; advised bedtimes^70,55^ or the order to stay in bed for a minimum of 7.5 hours per night^57^. One study directly started with sleep restriction or extension^67^.

## SUMMARY AND DISCUSSION

Although different methodological tasks and measurements have been summarized above, the measurements and results were discussed in accordance to the introduced multifaceted model of emotion regulation^24^.

Regarding neurophysiological processes, research utilizing EEG measurements^49,51^ supported the finding that sleep has a positive influence on the perception of emotional stimuli, as well as on the processing of automatic responses, due to an increase of neurological activity^49,51^. The reported results regarding cortical activity are congruent to comparable studies in adults^86^.

In contrast to adolescent’s^55^, children’s^53,54^ generation of a behavioral response to attention attracting neutral stimuli seem not to be affected by sleep manipulation. One explanation of the missing effect of sleep manipulation on attentional processes in children may relate to the small amount of sleep deprivation in children. It would be interesting to repeat the task by implementing longer periods of wakefulness to assess their consequences on children’s attention regulation processes. Regarding the results from research utilizing emotion eliciting stimuli, it could be concluded, that low sleep quality reduces the accuracy in processing emotional stimuli in adolescents^56^. Whereas studies including sleep manipulation utilize neutral stimuli, one study, concerning the influence of sleep quality on emotional processing, discriminated between the effect of neutral versus emotional stimuli. Unfortunately, the effect of either positive or negative emotional content of the stimuli was disregarded. Whereas recent research assessed attentional processes in an objective manner, future research should assess the influence of sleep deprivation as well as sleep parameters on children’s and adolescents’ use of attention regulation processes. Those processes according to Thompson (1994)^24^ encompass, e.g. the use of concrete strategies of conscious avoidance of emotion eliciting stimuli.

The effect of sleep deprivation on emotional reasoning is inconsistent to the effect of low sleep quality on it. Reddy et al. (2017)^57^ suggested that one night of sleep deprivation has no significant effect on reappraisal tactics and reasoning in adolescents, whereas a short sleep duration seemed to be linked to a decrease of emotional reasoning^58^. According to the results emotional reasoning seems not to be sensitive to sleep deprivation, whereas shortcomings in sleep quality cause lower performance in emotional reasoning. Regarding the operationalization of emotional reasoning, both studies made use of static pictures, differing in representing either emotion eliciting context^57^ or emotional facial expressions^58^. Unfortunately, in both studies, the emotion specificity of the results regarding the emotional reasoning did not report the differences in positive or negative reasoning distinctively. Future research should shed light on influence of longer periods of sleep restriction on emotional reasoning; consider reporting effect of sleep parameters on positive and negative reasoning about emotional states, and utilization of less static sources of stimuli, as short sequences of video clips with emotional content.

Results of studies concerning the physiological background of emotion regulation^49,60^ indicate a mutual dependence of emotional stimuli, physiological responses, and parameters of sleep. Findings support the idea, that rather than acting as a unity entity, emotion regulation and emotional responses emerge from interaction between automatic generating responses and cognitive processes, while both systems are sensitive for sleep. Beyond, future studies might wish to consider the implementation of behavioral ratings, as well as physiological and neuroimaging measures, to provide support to the body of literature regarding influencing factor of sleep on the association between physiological and emotion regulating processes in children and adolescents.

While focussing on coping, results of studies are supporting the assumption, that day and night sleep deprivation affects young children’s emotional coping competencies, whereas afternoon nap deprivation preserves increase use of maladaptive strategies in response to puzzle tasks^61,62^, as well as to stressing situations^63^. Findings of these observational studies are supported by findings from cross-sectional studies examining a longer night^64^ and day^65^ sleep duration to be associated positively to functional use of coping strategies. Another study found indirect, instead of direct effects of mild night sleep restriction on self-regulation strategies, mediated by children’s performance regarding response inhibition before being sleep restricted^53^. Therefore, future studies might wish to consider children’s’ coping predisposing factors as e.g. response inhibitation^53^ in more depth. Additionally, the incongruent finding of direct^61,63^ versus indirect^53^ effects of sleep manipulation on coping strategies may contribute to the difference in restricting day^61,63^ or night sleep^53^ in young children. Additionally, Berger et al. (2012)^62^ introduced the puzzle challenge with a “solving segment”, thus children finished the puzzle and were praised for their performance. How a previous successful event could be linked to self-regulation strategies, while performing the unsolvable puzzle task after restriction of night sleep is therefore a subject of debate. Furthermore, children’s reactivity when they were exposed to the unsolvable puzzle was significantly decreased after nap deprivation^61,62^. This may be an indication of reduced cognitive engagement and lowered motivation to retrieve information from the environment^87^.

Studies of affective response ratings with young children are with each other comparable to the result of greater negative responses to negative visual stimuli after nap-deprivation^62,66^; this is congruent with the results after nocturnal sleep deprivation in adolescents^67^. Discrepant to the results of lower positive responses on positive stimuli in toddlers^62^ was the result of greater positive responses towards positive pictures after nap-deprivation in young children^66^. Furthermore, ratings of negative affect were not influenced after sleep deprivation in school-aged children^54^. One explanation may relate to the selected stimuli, and their ratings of valence and arousal. The precise identification number of stimuli and the total ratings were not specified^54,62,66^ and cannot be consulted for discussion. Furthermore, two studies consulted additional objective measurements to rate subjects’ responses^66,67^, whereas another study used ratings by a human rater^66^, thus discrepancies in ratings can contribute to differences in evaluation of emotional responses. Finally, there was inclusion of an auditory stimulus; thus, the additionally activation of the auditory sense can have an influencing effect on the emotional response^66,67^. Future studies should investigate the listed reasons for incongruent results in more depth.

A decline in responses of subjective positive affect ratings on the positive and negative affect schedule (PANAS)^88^ was observable after two^68^ and also seven^55^ nights of sleep deprivation. Thus, different periods of nocturnal sleep restriction had no effects on the PANAS results. However, unlike in behavioral studies implementing the PANAS^57^, the effect on negative affect seemed to remain unchanged after sleep deprivation. Keeping in mind the subjective character of the PANAS, subjects may express test items representing a negative affect state, e.g. “guilty”, “scared” or “afraid” as irrelevant, thus negative affect stays unaffected^55^. Behavioral studies with additional activation of visual or auditory senses^57,67^ found sleep deprivation to increase ratings of negative affect stimuli and therefore support the presented explanation. In the case of this difference, future research should choose the implementation of different measures and stimuli to investigate the effect of sleep deprivation on affect states.

Whereas Baum et al. (2014)^69^ found no increase of the dimension of depression after partial sleep deprivation for four nights, Short and Louca (2015)^70^ reported that girls significantly, and boys instead of males marginally, reported an increase of the feeling of being depressed after a night of strict sleep restriction. In contrast to the other mood states assessed in the POMS^89^, depressed mood seemed not that sensitive to moderate sleep deprivation^69^ than to strict sleep deprivation^70^. Secondly, the items of the POMS depressive subscale are comparable to those of the negative affect states in the PANAS, thus, they may be valued as irrelevant and consequently depressive mood seemed nearly unaffected. However, female’s sensitivity regarding depression and anxiety following experimental strict sleep deprivation was only reported by Short and Louca (2015)^70^. This is in line with the results of a longitudinal study; girls with early sleep problems displayed more anxiety-depressive symptoms in preadolescence^82^. These findings demonstrate interrelatedness between the female gender and the sensitivity for problematic or restricted sleep. Taken together, these results suggest significant effects of sleep loss on subjective affect and mood states, but some striking results regarding sleep loss and indices of depression. Furthermore, the influence of female gender needs to be clarified in future research.

Due to the results, a mutual association between mood dimensions and sleep quality can be assumed^71,72^. These results are also supported by observational and behavioral studies in young children^62,63^ and adolescents^67^. Regardless of their diligence in the selection of instruments and sampling procedures, these studies cannot meet the aim of giving insight into long-term development of affect and sleep, due to its cross-sectional character.

Differences in the effect of sleep manipulation on social emotion regulation behavior are also somewhat striking. These differences can be explained with reasons, e.g. the deviating moment of assessment of social emotion regulation behavior as well as by different schedules of sleep manipulation. Whereas participants in the study by McMakin et al. (2016)^67^ were tested after restricting their sleep to respectively four and two hours on two consecutive nights, participants in the study by Raynolds (2017)^73^ were investigated after extending their sleep to one additional hour for five consecutive nights. Another explanation might contribute to the analysis’s outcome measures. Methodologically, McMakin et al. (2016)^67^ also assessed facial expressions, as well as verbal content but, in contrast to Raynolds (2017)^73^, the two composite scores were calculated by averaging one summary score. The question about the effects of sleep restriction on respectively affective facial expressions and language remains open in the study by McMakin et al. (2016)^67^. Additionally, differences between human rating and computer results of these measurements in the context of sleep manipulating studies have not been conducted yet. Future studies should investigate the listed reasons for incongruent results in more depth.

The finding of moderating effect of sleep problems on the association of a child’s early negative emotionality and later internalizing behavior must be considered in a critical light of methodological limitations. Negative emotionality was assessed when children were 6 months of age on a 15 minutes’ interaction. Biasing factors such as mood, representing the mental state that temporarily predisposes a person to act to a variety of events^90^, were not controlled. Whereas the longitudinal association of negative emotionality, sleep problems and behavior must be given more attention in future research, longitudinal research should also implement assessment of the variables at more frequent points of measurement to detect their individual development as well as their mutual associations. Similar points of criticisms are given to Gruber et al. (2012)^77^. Even if the children’s and adolescents’ emotion regulation behavior is affected by sleep loss, according to subjective measurement, this result is one-dimensional and can be biased by un-controlled day-to-day influences in their environment. Thus, future research should be interested in the implementation of more objective measurements and task paradigms^67,73^, to assess the effect of sleep loss or sleep extension on regulatory behavior in the school or daily-life context.

Results from longitudinal research provide inconsistent results regarding the mutual developmental pathways of sleep and emotion regulation^81,82^. Reasons for these striking results do not contribute to the measurement of sleep problems, because the child behavior checklist (CBCL)^91^ was employed and the sources of information were the subjects’ mothers in both cases. However, statistical analyses differed; whereas Gregory and O’Connor (2002)^81^ made use of hierarchical regression analysis, Foley and Weinraub (2017)^82^ proceeded through seven stages for testing longitudinal cross-lagged panel models, which is a more sensitive analysis because of the control of autoregressive effects and covariation among variables^91,92^.

An association between development of emotional problems due to early sleep problems can be assumed^81,82,85^, whereas the reverse association is still to debate^81,82^. According to Wang et al. (2019)^83^ the chance to rehabilitate from emotional problems increased in consequence of improvement of sleep problems.

On the one hand experimental sleep manipulation studies have the power to demonstrate cause and effect relationships because of its strict sleep schedules^55,68,69^, a few studies reduced the explanatory power due to unrealistic and extreme^70^ or short-term^67^ sleep manipulations. On the other hand, correlational studies have focused on natural circumstances, but have not allowed for cause-effect conclusions^64^. While the present review sums up results as well as the methodological approach the findings from experimental nap and sleep manipulation converge with each other and it could be assumed that real-world associations between sleep and emotion regulation reflect a true cause and effect association.

In sum, the reliance on self-, parental-, and teacher reports rather than objective measures of sleep and emotional constructs represented a considerable limitation in these longitudinal studies. Furthermore, there is still the request of research to address the temporal development of problematic sleep and behavioral regulation problems, and the influencing conditions under which sleep and regulation may develop into a negative cyclical pattern. The clarification of the hen egg problem remains a challenge for future studies.

### Strengths and limitations

As demanded^2^, this review is the first attempt to identify the relationship between the impact of sleep manipulation as well as sleep parameters on different emotion regulation processes across the youth upcoming from a developmental, multifaceted model of emotion regulation. Studies methods and instruments are aggregated and serve as a structure for future research that is interested in the assessment of sleep and affect regulation in a young population. According to our selection criteria, studies addressing children and adolescents suffering from diagnosed mental or sleep disorders, chronic illness, or in special medical circumstances were excluded. Therefore, it should be kept in mind that results are representative only for healthy and normally developed children and adolescents.

Studies in infants or toddlers rely on parental report^54,64,65^ and observational methods^62,76^ in large part, because very young children have considerable limitations reporting intrapersonal emotional experiences^17^. It is important to consider, that reliance on parental report might lead on to misinterpretation and overestimation of the results due disregarding influence of potential parental covariates on their statements^17^.

Furthermore, as research has shown, studies assessing emotion regulation processes under controlled conditions increases the probability of activating special emotions^39^. A distinction of naturalistic or laboratory settings is essential to be able to assume that upcoming emotional affect regulate behavioural expressions, as a successful event can enforce a children’s resilience through frustrating situations^62^ or that emotional affect can be regulated e.g. adolescent’s positive reappraisal reduced their negative affect states^57^. Thus, disregarding the interference between a constructed setting and emotional responses prevent an integral understanding of the context of emotion regulation.

With respect to the implementation of emotional images from the IAPS^54,62,66^ the precise identification of chosen images was not specified in all cases; studies that implement images with increasingly gradients may exaggerate the effects of sleep deprivation and while IAPS is the source of stimuli in all studies, affective reactivity may differ in accordance with specific images, so methodological diligence is demanded for the transparency and validity of the research.

It must be stated that studies differ in their techniques of analysis as well as in data generation^62,73^. When comparing the studies, a change in a sum score in the affect relating variables^62^ does not have that interpretability as its individual reactivity to manipulated sleep^73^. To avoid a generalizing conclusion of the associations between sleep and affect, these differences and analytical details must be considered during the interpretation of results. Additional studies which did not report the significance of group differences are also less representable^77^.

However, some measures of implemented subjective affect^57^ were not as sensitive to sleep deprivation as others^77^, because the test items may not be relevant to the sample. Furthermore, assessment an infant’s manageability by one item might be somewhat insufficient^64,65^. Thus, when preparing research, it is necessary to be careful when verifying the suitability of the instruments for measurement in the target group.

### Key limitation

A key limitation of the studies including sleep manipulation was that there were only two sleep or nap conditions. The contrast between the experimental groups of sleep or nap restriction and unmanipulated condition offers evidence of causation, but does not considering the individual preferences and sleep habits of participants. This negotiation may lead to exaggeration or underestimation of the effects of sleep or nap deprivation on emotion regulation outcomes. While the relationships between sleep manipulation and outcome measures of emotion regulation are debated, it seems important to attempt to identify the threshold at which sleep or nap deprivation affects emotions in young children and adolescents. Therefore, future experimental research should vary in sleep duration, as well as in bed and raise times to assess potential differences in impact on emotional outcomes.

### Future directions

According to the remarks of this systematic review, the implications for future research are related to: 1) diversity of measurements and sources, 2) variety of task characteristics and procedure, 3) requirements for longitudinal research, and 4) gender vulnerabilities of sleep and affect.

### Diversity of measurements and sources

*Affect processes.* Affective processing has several elements affected by sleep, such as the generation of affect states^57^, the duration of emotional expressions^62^, and emotional response behavior^63^. Therefore, individual measures may provide an incomplete picture of the interconnection of sleep and affect. Emotion regulation is complex, and only two studies assessed cortical activity, which contributes towards emotion regulation behavior^49,51^. Physiological as well as neuroimaging measurements provide information for the neural and physical responses while recepting an affective stimulus. Parental or teacher reports in toddlers and children, or self-report can be implemented to assess subjective measurements of affective and emotion states. Keeping in mind, that implementation of a specific questionnaire itself can have influence on the result^69,70^. It is the same for objective ratings and observations by humans, as well as by computer, to provide information on the emotion regulation, and emotion responding behaviors. To investigate the influencing effect of sleep on the whole process of affect processes, behavioral, physiological, neuroimaging, and subjective measurements need to be implemented.

*Sleep assessment.* This review provides the impression that, especially, longitudinal studies evaluate subjective sleep data more frequently than objective sleep data. Therefore, objective data, such as actigraphy should additionally be implemented in the research standard, due to the higher quality of data^93^. However, the use of actigraphy in infants or toddlers faces problems of appropriate algorithms^94^ to detect real night awakenings and, therefore possibly making use of behavioral sleep observation.

### Variety of task characteristics and procedure

*Stimuli presentation and sense activation.* Additionally, it was reported that there are differences in the presentation of the stimulus (number, duration, rating-interval, and static image)^62,66,67^. This alone also can have an impact on the emotional response. To investigate the effect of these differences, experimental tasks could vary in the time of stimuli presentation or in the duration of the interval between stimuli. Additionally, there was evidence that emotional response ratings are sensitive to the content category of stimuli (neutral, negative, and positive). For precise results, future studies should always report differences, as well as missing difference in their subject’s responses towards neutral, positive, or negative stimuli. Furthermore, the activation of an additional sense during affect processes can also have an effect on the responses to the stimuli^66,67^. Differences in affect responses after stimulating different senses, and their sensitivity to sleep loss or sleep parameters may be interesting to investigate in the future. The relationships between different types of stimuli and presentation appear important to take into account.

*Individual condition before assessment.* Neural activity in response to emotional stimuli was affected by different sleep conditions^49^, and there is evidence that speed of generation of behavioral responses increased through enhanced cortical activity^51^. As such, the individual condition of brain activity could exert an additional effect on responses towards emotional stimuli. The role of psychological constitution before assessment seems to have an influential effect on the affect responses^62,90^. According to the finding from observational studies, the idea arose that the implementation of positive, successful events can enhance emotion regulation capacity during frustrating daily-life situations after sleep loss has emerged^62^. As such, future research aimed at these “mood manipulations” could give insight into the biasing influence of temporal mood, as well as subject’s internal factors as e.g. temperament^65^ on sensitivity to actual sleep loss and affective responses and, additionally, help to identify factors of resilience and better emotion regulation.

*Sleep manipulation.* In accordance with the results, individuals vary in their reactivity to sleep or nap-deprivation, and in their responses to different types of emotional measures. It is indisputable that the schedules of sleep deprivation alter in accordance with the subject’s age, but there were also differences in scheduling of sleep deprivation and manipulation within the group of toddlers^53,62^, school-aged children^59^, and adolescents^66,67^. These factors suggest that the perceived reactivity of sleep restriction on constructs of emotion regulation cannot be explained by performance observation or ratings alone; the procedure of sleep manipulation and the amount of sleep deprivation could also affect the gradient of reactivity. It is important to take into account the different schedules and durations of sleep loss, as well as their dose response costs on emotional processes.

### Requirements for longitudinal research

*Methodological demands.* Longitudinal studies that examine the role of sleep parameters in the context of affect processes often limit the lack of assessment of affective and sleep related constructions simultaneously^76^. Research concerning the temporal development of the inter-relationship of sleep and affect, as well as influencing conditions under which sleep and affect regulation may develop into a negative pattern is underrepresented. Additionally, changes in the development of physiological responses to sleep deprivation in the context of the processing of emotional stimuli in children and adolescents should be considered in the future. The methodological challenge when planning longitudinal research contributes to the limited set of appropriate experimental tasks, because such a task needs to develop with the participant’s age. Therefore, within the scope of its variability of age-appropriate images of the IAPS, it seems to be a good option in regards to the affect stages, but in the scope of its increasing task demands, the Balloons task^95^, especially, allows the implementation of the same measure across a wide range of ages. Conducting such demanding research may be important for the understanding of the development of serious sleep, as well as affective problems and their mutual association.

*Longitudinal outcomes.* To bring back Thompson’s assumption, arguing that individuals construct their life-settings whilst taking into account the expected emotional load^24,43^, it is remarkable that none of the included studies was focused on the long-term consequence of sleep problems in childhood or adolescence for the later emotional load of their life-time settings, burdens or responsibilities. As reported in many studies there might be an association between healthy sleep early in life on better later mental health, resilience or performance^8,9,11^, early sleep could be expected to have impact on creation of the life setting, but to what extent remains open for future research. Evidence of this association could emphasize the importance of detection and treatment of problematic sleep during childhood and adolescence.

### Vulnerability of gender to sleep and affect

Few studies have investigated the role of gender in the subjective report of noticeable behavior problems and less social competence^82,84^. The female gender was found to react with more emotional reactivity to problematic sleep and loss of sleep^70,84^. Possible reasons are for example gender related differences in hormonal regulation^96^. Furthermore, adolescent males are less willing to report and confess emotions such as depressive mood in their self-report^97^. Explanations of these findings were not investigated in the context of sleep and affect studies. Therefore, care needs to be taken in the interpretation of outcomes of this study and seen as hints towards differences, which should be evaluated in upcoming research.

## CONCLUSION

Sleep seems to be associated to each level of emotion regulation processes. Sleep plays a role in the ongoing regulation of emotional arousal from infancy to adolescence. Affect processes in childhood and adolescents seems to be negatively affected by sleep problems or sleep deprivation, whereas prolonged sleep is a predictor for fewer behavioral problems, less negative affect states and better affect regulation. Sleep promotes sensory processing of emotional stimuli, and is most obvious in neuroimaging and physiological measures. Sleep deprivation impairs affect processing, with apparent results in subjective and objective measurements of affective reactions towards visual emotion eliciting stimuli. Observational studies suggest that sleep enhances the duration and strength of positive emotions towards successful events, as well as the ability for emotional reasoning, behavioral response, and regulation.

In general, associations of sleep and levels of affect in this review are restricted to young subjects. Furthermore, the types of measurements, tasks and data analyses may also have some influence on the results. Future studies should be based on more diverse measurement of affect and sleep; they should vary in their stimuli presentation and sense activation. Further, they should implement mood manipulation to investigate the mutual associations in some detail. The consideration of gender differences in affect processes and sleep are also important. Additionally, the role of different amounts of sleep loss during sleep deprivation requires clarification. Detailed and well-planned longitudinal research concerning temporal development of sleep and affect is needed. Following from the results, less sleep can be assumed to have a negative impact on affect processes, and is associated with notable short- and long-term psychological problems; therefore, understanding the relationships between affect processes and sleep is a central assignment for future research.

Regarding the practical implications the results indicate a direct relationship between sleep and emotional functioning in a young population. Therefore, prevention and intervention programs should increase sensitivity for sleep problems and its recognition. Moreover, psychoeducation regarding sleep and its positive and negative consequences among parents, children, adolescents, and health care professionals might provide faster recognition and a better opportunity for successful treatment of sleep irregularities and therefore prevent chronification and potential psychopathological consequences.

**Table:** 

Practice points	
• Sleep supports processing of emotional stimuli, obvious in physiological and physiological measures.	
• Sleep deprivation impairs affect processing, measured by subjective and objective measurements of affective reactions towards visual and auditory emotion eliciting stimuli.	
• Longer sleep duration, as well as undisturbed instead of quite sleep enhances the experience of positive emotions, as well as the capacity of emotional reasoning, behavioral responding and regulation.	

**Table:** 

Key research agenda
• Include diverse measurements of affect and sleep, and examine the presentation of stimuli and the activation of the senses.
• Be highly sensitive while choosing the methods of assessment and manipulation of sleep and emotion regulation.
• Differences in results have to be interpreted with regard to the used statistical analysis.
• Explicitly discriminate between assessment of emotion activation and emotion regulation.
• Investigate the role of different amounts of sleep loss during sleep deprivation on task performance, and involve the possible interference between a constructed setting and emotional responses in the interpretation of results.
• Assess the temporal development of sleep and emotion to clarify their mutual association during childhood and adolescence.
• Investigate influencing conditions under which sleep and affect regulation develop into a negative pattern.
• Implement and develop objective tests with age-appropriate and variable task demands for longitudinal research.
• While utilizing report of others, pay attention to possible confounders.
• Identify confounding intra-individual factors of better emotion regulation.
• Acknowledge of gender as an influencing factor.

## APPENDIX

**Table 1:** Sleep manipulation protocols of studies concerning infants and toddlers (n=5).

Study	Days of stabilization period	Sleep duration/ bedtime		Day(s) of manipulation period	Sleep condition	Sleep duration/ bedtime		Point of measurement	Content of measurement
		Night	Day			Night	Day		
Berger (2012)^62^	1-5	20:00-07:00	12:30-14:00	6	Nap restriction	20:00-07:00	-	15:00	Emotional assessment, video recorded (30min): • 11 emotion eliciting pictures • Puzzle task
	6-11	20:00-07:00	12:30-14:00	11	Normal sleep	20:00-07:00	12:30-14:00	15:00	
Cremone et al. (2017)^52^	0	ns	ns	1	Nap restriction	ns	-	≈15:00	Emotional attention bias • Dot probe task
	0	ns	ns	1	Normal nap	ns	13:00-15:00	≈15:00	
Han (2014)^66^	1-7	11-12hrs/day		8	Nap restriction	ns	-	ns	Emotional assessment (11min) • 32 visual and auditory stimuli pairings
	9-11	11-12hrs/day		12	Normal nap	ns	Nap option in lab	ns	
Miller (2015)^61^	1-5	20:00-07:00	12:30-14:00	6	Normal sleep	20:00-07:00	12:30-14:00	15:00	Challenge task, video recorded: • Puzzle task
	6-11	20:00-07:00	12:30-14:00	11	Nap restriction	20:00-07:00	-	15:00	
Schumacher (2017)^53^	1-5	20:00-07:00	12:30-14:00	6	Normal sleep	20:00-07:00	12:30-14:00	10:00	Challenge task, video recorded: • Go/no go task • Puzzle task
	6-10	20:00-07:00	12:30-14:00	11	Sleep restriction	23:00-07:00		10:00	

Abbreviations: ns = Not specified.

**Table 2:** Sleep manipulation protocols of studies concerning children (n=2).

Study	Days of stabilization period	Sleep duration	Day(s) of manipulation period	Sleep condition	Sleep duration/ bedtime	Point of measurement	Content of measurement
Gruber (2012)^77^	1-5	Individual bed/ sleeptimes Randomization after stab. period	6-10	Sleep extension	1hr earlier than usual	• Last day of baseline and manipulation period	• The Connors’ global index-teachers with subscale “emotional lability” and “restless-impulsive behavior”
			6-10	Sleep restriction	1hr later than usual		
Vriend (2013)^54^	1 week	Individual bed/ sleeptimes Randomization after stab. period	4 nights	Sleep extension	1hr earlier than usual	• Baseline in lab • In the morning after last night of sleep restriction • In the morning after last night of sleep extension	Assessment battery (90-120 min): Parents: • Parents questionnaires Conners’ parent rating scale-revised • Emotion questionnaire Children: • Child’s pictorial sleepiness scale • Digit-span task • Finger windows task • Attention network test-interaction • Children’s colour trial test • Math fluency task • Emotion questionnaire for children
			4 nights	Sleep restriction	1hr later than usual		

**Table 3:** Sleep manipulation protocols of studies concerning adolescents (n=7).

Study	Days of stabilization period	Sleep duration	Day(s) of manipulation period	Sleep condition	Sleep duration/ bedtime	Point of measurement	Content of measurement
Baum (2014)^69^	5 nights	Individual bed/ sleeptimes				• Baseline assessment • Assessment after last night of SR • Assessment after last night of SE	Parents: • Vanderbilt assessment scale • Emotion control subscale of the BRIEF Adolescents: • Profile of mood states Emotion control subscale of the BRIEF
	2 nights	Individual bed/ sleeptimes	4 nights	Sleep restriction	6.5hrs in bed		
	2 nights	Individual bed/ sleeptimes	4 nights	Sleep extension	10hrs in bed		
Dagys et al. (2012)^68^	5 nights	Individual bed/ sleeptimes Randomization after stab. period	1 night at home	Sleep restriction	In bed for 6.5hrs		
			2nd night in lab		03:00-05:00	22:00; 00:00; 02:00; 03:00; 05:00; 08:00	• Positive and negative affect schedule-children
			2 nights at home	Sleep extension	8.5hrs in bed	08:00 or 10:00 in lab	
Lo et al. (2016)^55^	3 nights	23:00-08:00	7 nights	Sleep restriction	01:00-06:00	• Assessment every day at 10:00; 15:00; 20:00	Cognitive performance test battery (25min): • Karolinska sleepiness scale • Sustained attention to response task • Symbol digit modalities test • Verbal 1- and 3 back tasks • Mental arithmetic test • Positive and negative affect schedule • PVT
			7 nights	Normal sleep	23:00-08:00		
				
McMakin (2016)^67^ Study protocol 1	-	-	2 nights	Sleep restriction	01:00-05:00	Assessment of affect three times during day one and one time in the morning after the 2^nd^ night	• PSG • Visual analogue scales • Auditory valence identification task, pupillography
			2 nights	Sleep extension	22:00-08:00		
McMakin (2016)^67^ Study protocol 2	-	-	2 nights	Sleep restriction	1st night 02:00-08:00 2nd night 02:00-04:00	Assessment of affect three times during day one and one time in the morning after the 2^nd^ night	• PSG • Visual analogue scales • Auditory valence identification task, pupillography • Peer conflict task in the afternoon of day 2
			2 nights	Sleep extension	22:00-08:00		
			4 nights	Sleep extension	Go to bed as usual and stay in bed for 9.5hrs		
**Stud**y	**Days of stabilization period**	**Sleep duration**	**Day(s) of manipulation period**	**Sleep condition**	**Sleep duration/ bedtime**	**Point of measurement**	**Content of measurement**
Raynolds (2017)^73^	1-7	Individual bed/ sleeptimes Randomization after stab. period	5 nights	Normal sleep instead of unmanipilated	Go to bed as usual	Assessment in lab between 16:00 and 18:00 after last night of sleep manipulation	Computerized task battery, videotaped Naturalistic online social interaction task • Frustrating PC game Manipulated online social interaction task
			5 nights	Sleep extension	1hr earlier than usual		
Reddy et al. (2017)^57^	1-6	Minimum of 7.5hrs in bed	4 nights	Sleep restriction	2hrs later than usual and only 4hrs in bed	Assessment in lab at 12:00 after last night of sleep manipulation	• Kiddie schedule for affective disorders and schizophrenia for school-age children-present and lifetime version • BEARS sleep screen • Positive and negative affect schedule-children • State-trait anxiety inventory for children • Epworth sleepiness scale • Emotion reactivity and regulation task based on the international affective pictorial system (16min, 40 trials)
			4 nights	Sleep extension	Go to bed as usual and stay in bed for 9.5hrs		
Short and Louca (2015)^70^	1 week	22:00-07:00	2 nights	Normal sleep	22:00-08:00	09:00; 11:00; 13:00; 15:00; 17:00; 19:00	Neurobehavioral test battery: • Psychomotor vigilance task • Digit symbol substitution task • Profile of mood scale-short form • Karolinska sleepiness scale
			1 night	Sleep restriction	08:00-20:00 (36hrs awake)	Every 2 hours testing	
